# Single fibre cytoarchitecture in ventilator-induced diaphragm dysfunction (VIDD) assessed by quantitative morphometry second harmonic generation imaging: Positive effects of BGP-15 chaperone co-inducer and VBP-15 dissociative corticosteroid treatment

**DOI:** 10.3389/fphys.2023.1207802

**Published:** 2023-06-27

**Authors:** Sofia Mnuskina, Julian Bauer, Anette Wirth-Hücking, Dominik Schneidereit, Stefanie Nübler, Paul Ritter, Nicola Cacciani, Meishan Li, Lars Larsson, Oliver Friedrich

**Affiliations:** ^1^ Department of Chemical and Biological Engineering (CBI), Institute of Medical Biotechnology, Friedrich-Alexander University Erlangen-Nürnberg, Erlangen, Germany; ^2^ Department of Physiology and Pharmacology, Karolinska Institutet, Stockholm, Sweden; ^3^ Department of Clinical Neuroscience, Clinical Neurophysiology, Karolinska Institutet, Stockholm, Sweden; ^4^ Viron Molecular Medicine Institute, Boston, MA, United States; ^5^ Muscle Research Center Erlangen (MURCE), Friedrich-Alexander University Erlangen-Nürnberg, Erlangen, Germany; ^6^ School of Medical Sciences, University of New South Wales, Kensington Campus, Sydney, NSW, Australia

**Keywords:** critical illness, diaphragm dysfunction, second harmonic generation, BGP-15, quantitative morphometry

## Abstract

Ventilator-induced diaphragm dysfunction (VIDD) is a common sequela of intensive care unit (ICU) treatment requiring mechanical ventilation (MV) and neuromuscular blockade (NMBA). It is characterised by diaphragm weakness, prolonged respirator weaning and adverse outcomes. Dissociative glucocorticoids (e.g., vamorolone, VBP-15) and chaperone co-inducers (e.g., BGP-15) previously showed positive effects in an ICU-rat model. In limb muscle critical illness myopathy, preferential myosin loss prevails, while myofibrillar protein post-translational modifications are more dominant in VIDD. It is not known whether the marked decline in specific force (force normalised to cross-sectional area) is a pure consequence of altered contractility signaling or whether diaphragm weakness also has a structural correlate through sterical remodeling of myofibrillar cytoarchitecture, how quickly it develops, and to which extent VBP-15 or BGP-15 may specifically recover myofibrillar geometry. To address these questions, we performed label-free multiphoton Second Harmonic Generation (SHG) imaging followed by quantitative morphometry in single diaphragm muscle fibres from healthy rats subjected to five or 10 days of MV + NMBA to simulate ICU treatment without underlying confounding pathology (like sepsis). Rats received daily treatment of either Prednisolone, VBP-15, BGP-15 or none. Myosin-II SHG signal intensities, fibre diameters (FD) as well as the parameters of myofibrillar angular parallelism (cosine angle sum, CAS) and in-register of adjacent myofibrils (Vernier density, VD) were computed from SHG images. ICU treatment caused a decline in FD at day 10 as well as a significant decline in CAS and VD from day 5. Vamorolone effectively recovered FD at day 10, while BGP-15 was more effective at day 5. BGP-15 was more effective than VBP-15 in recovering CAS at day 10 although not to control levels. In-register VD levels were restored at day 10 by both compounds. Our study is the first to provide quantitative insights into VIDD-related myofibrillar remodeling unravelled by SHG imaging, suggesting that both VBP-15 and BGP-15 can effectively ameliorate the structure-related dysfunction in VIDD.

## 1 Introduction

Critical illness and treatment of critically ill patients in intensive care units (ICU) has been steadily increasing over decades, e.g., about 15% between 2000 and 2009 in the United States (Wallace et al., 2014). The Society of Critical Care Medicine states more than 5 M. United States citizens being admitted to ICUs annually, 20%–40% of which receive supportive mechanical ventilation (https://www.sccm.org/Communications/Critical-Care-Statistics). Apart from the costs afflicted with treatment of the underlying disease, common neuromuscular sequelae of ICU treatments, i.e., critical illness neuropathies, myopathies and combinations thereof, usually prolong ICU stays and interventions, are associated with complications and may worsen patient outcome (Latronico et al., 2005; Hermans et al., 2014). In particular, the COVID-19 pandemic has seen a vast increase in ICU admissions of patients with severe respiratory disorders, such as acute respiratory distress syndrome, that required invasive mechanical ventilation (Gonzalez et al., 2022). As a consequence, studies have documented a definite critical illness myopathy (CIM) prevalence of more than 50% in such critically ill COVID-19 patients (Rodriguez et al., 2022).

One hallmark of CIM is a progressive preferential myosin loss of the sarcomeric contractile proteins due to transcriptional downregulation of myosin synthesis and increased myosin protein degradation as reflected in a drop of myosin:actin ratios by 50% within 2 weeks of ICU treatment in patients (Cacciani et al., 2022). The complete mechanical silencing of skeletal limb muscle in ICU patients and ICU-animal models was therefore, suggested to lead to a compromised ‘quality’ of contraction beyond the reduction in single fibre cross-sectional area (CSA) as seen by a marked drop in specific, CSA-normalized forces (Ochala et al., 2011a; Llano-Diez et al., 2012; Friedrich et al., 2015; Corpeno Kalamgi et al., 2016; Friedrich et al., 2017). Interestingly, it has also become apparent that different muscles are differentially affected by the ICU-treatment with severe myosin proteolysis seen in limb muscles but only minor and delayed myosin degradation in cranio-facial muscles (Akkad et al., 2014).

The diaphragm also expresses profound muscle weakness through ongoing mechanical ventilation, termed ventilator-induced diaphragm muscle dysfunction (VIDD), that results in delayed weaning and poor outcome (Hermans et al., 2010; Larsson and Friedrich, 2016). In sharp contrast to limb muscles of ICU patients (Larsson et al., 2000; Llano-Diez et al., 2012) and experimental animal models (Norman et al., 2006; Ochala et al., 2011a), the myosin:actin ratios in diaphragm muscle were mostly preserved throughout the observation period of up to 14 days in a rat ICU-model, albeit a significant and progressing drop in specific force by already 25% within the first 4 days of mechanical ventilation (MV) (Corpeno Kalamgi et al., 2016). This marked decline in contractile performance also preceded the development of single fibre atrophy with preserved CSA until day 4 but declining to 50% CSA alongside with ∼30% residual specific force after 14 days of MV (Corpeno Kalamgi et al., 2016). Preservation of myosin:actin ratios and CSA also applied to diaphragm fibres investigated in a piglet-ICU model with MV performed for 5 days compared to controls. Those also did not change by combining with additional stressors, like sepsis corticosteroids (CS) or neuromuscular blockade (NMBA) treatments despite decreased specific CSA-normalised forces (Ochala et al., 2011a).

One of the key differences between limb muscles and diaphragm in immobilised ICU patients is that the diaphragm is kept under mechanical load and stresses through the controlled mechanical ventilation regime which has been found to lead to oxidative stress, post-translational protein modifications (PTMs) and intracellular lipid accumulation within days of MV in the rat ICU-model (Corpeno et al., 2014). Reactive oxygen species (ROS), induced by mechanical ventilation, have been shown to target mostly insoluble proteins for oxidation and degradation (Zergeroglu et al., 2003). The discrepancy between early oxidative stress but preserved sarcomeric protein content and preserved sarcomere patterns in electron micrographs of diaphragms from rats mechanically ventilated for up to 10 days suggests an impaired quality of contraction that may be explained by a bioenergetics failure given that abnormal structural changes of diaphragm mitochondria coincided with MV duration (Salah et al., 2016). However, structural integrity of the myofibrillar lattice in single fibres is a major determinant of integrated force production of the poly-myofibrillar array that has not been studied yet in VIDD as a potential cause of weakness.

The concept of ‘quantitative morphometry’ includes assessment of relative orientation and registry of adjacent myofibrils along the fibre length to determine angular deviations in 3D. This can be label-free obtained from single muscle fibres up to thick muscle sections using multiphoton Second Harmonic Generation (SHG) imaging to obtain structural myofibrillar lattice parameters of Cosine Angle Sum (CAS) and Vernier Density (VD) as a predictor of structure-related muscle weakness (Schneidereit et al., 2018). Thus, single fibre force detriments can be delineated from optical information originating from subcellular structures, like sarcomeric myosin-II (Both et al., 2004; Friedrich et al., 2010).

To address the hypothesis that controlled mechanical ventilation and neuromuscular blockade ‘*per se*’ may lead to an early disorder of the myofibrillar array preceding fibre atrophy as an ultrastructural correlate of the early blunting of specific force in VIDD, we applied SHG imaging and quantitative morphometry to single diaphragm fibres obtained from the rat ICU-model at the beginning and following five and 10 days of MV + NMBA. The rats undergoing the MV + NMBA treatment were healthy, i.e., there were no additional confounding pathologies induced, such as sepsis, during the course of the study. Therefore, our approach reflects a setting to study ultrastructural changes in VIDD.

In addition, we were also interested in whether myofibrillar lattice disarray induced during MV + NMBA could be targeted for prevention or amelioration of ultrastructural changes by pharmacological intervention during the ICU treatment. As diaphragm oxidative stress may be a major trigger for contractile protein pathology associated with reduced heat shock protein (HSP) activation (Ogilvie et al., 2016), the HSP72 chaperone co-inducer BGP-15 was chosen. Targeting HSP72, either by BGP-15 intervention (Ogilvie et al., 2016; Salah et al., 2016) or endurance exercise before MV (Smuder et al., 2012; Smuder et al., 2019) has been shown to restore force generating capacity of diaphragm muscle fibres and to ameliorate VIDD.

Another promising compound is vamorolone (VBP-15), a new class of dissociative glucocorticoids able to selectively activate anti-inflammatory pathways and protect from activation of muscle proteolytic pathways (e.g., MuRF1, atrogin-1) such as normally seen with other corticosteroids (e.g., Prednisolone) (Akkad et al., 2019). VBP-15 has proven safe in first Phase 1 human clinical trials (Hoffman et al., 2018) and was potent in reducing atrophy and weakness in the rat ICU-model for 5 days of ICU treatment and drug treatment (Akkad et al., 2019).

We provide a first explanation to the decline in specific force in VIDD during ongoing mechanical ventilation and neuromuscular blockade that is reflected by myofibrillar disarray in single fibres already after 5 days. We also report beneficial effects of applying chaperone co-inducer BGP-15 or dissociative glucocorticoid VBP-15 on myofibrillar disarray in VIDD.

## 2 Methods

### 2.1 Rat ICU-model and pharmacological interventions

**FIGURE 1:**
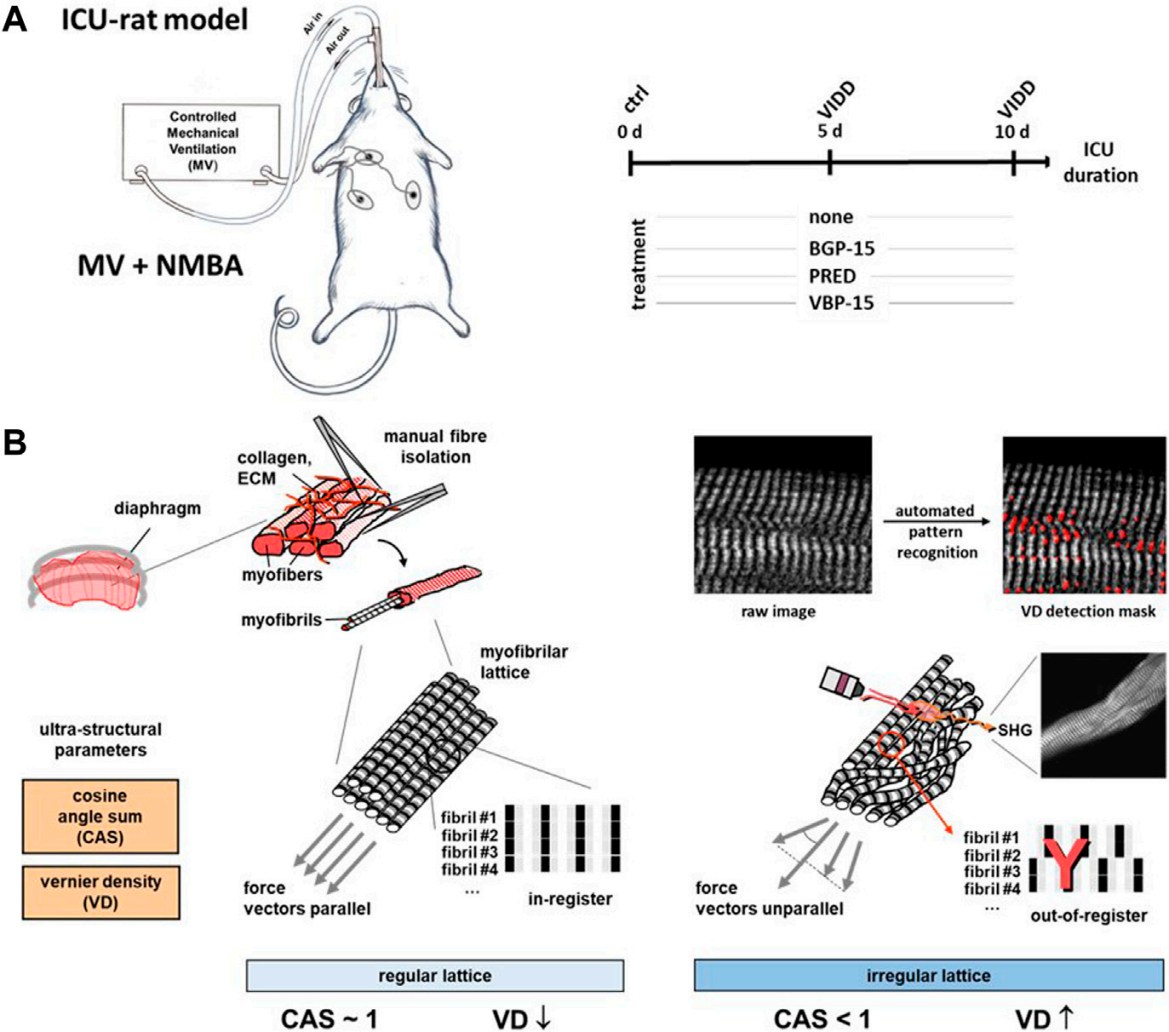
Experimental workflow of the rat ICU model, pharmacotherapy and diaphragm fibre multiphoton morphometry for ventilator-induced diaphragm dysfunction (VIDD). **(A)**, schematic workflow of the differential treatment regimes in a mechanical ventilation (MV) and neuromuscular blockade (NMBA) rats kept under ICU conditions being either subjected to a 10 days course of no drug treatment (‘none’), Prednisolone (PRED), BGP-15 or VBP-15. At days 0 (‘ctrl’), 5 and 10, diaphragm muscle was harvested for subsequent single fibre isolation and label-free multiphoton Second Harmonic Generation (SHG) imaging, followed by quantitative morphometry for SHG signal intensities, fibre diameters and the myofibrillar architecture alignment as represented by the parameters ‘cosine angle sum’ (CAS) and ‘Vernier densities’ (VD) **(B)**. In healthy single fibres, regularly aligned myofibrillar lattice is characterised by a CAS ∼1 and low VD values, while lower CAS and increasing VD indicate myofibrillar twists and tilts as well as out-of-register shifts.

The objective of this study was to evaluate effects of BGP-15, Prednisolone and vamorolone on diaphragm muscle structure. 26 healthy adult female Sprague-Dawley rats weighing 314 ± 6 g were included in this study. The rats were randomly divided into one control group (n = 4, day 0) and four treatment groups exposed to controlled mechanical ventilation, neuromuscular blockade and deep sedation for 5 or 10 days with no drug treatment (ICU group ‘none’, n = 6), with Prednisolone (ICU group PRED, n = 5), with vamorolone (ICU-group VBP-15, n = 5), and BGP-15 (ICU group BGP-15, n = 6) (Figure 1A). The effects of these interventions on regulation of muscle contraction and muscle fibre size have been presented in previous studies (Salah et al., 2016; Akkad et al., 2019). At the end of the experimental period, rats were euthanized and samples were collected for analyses. Controls (‘ctrl’) underwent the same ICU interventions as the experimental rats, but without the administration of α-cobratoxin and were euthanized within 2 hours of the initial surgery (labelled ‘0 day’).

Experimental details on sedation, anaesthesia, surgery (intravenous and intra-arterial catheterization of *V. jugularis* and *A. carotis*), mechanical ventilation, protein and fluid balance, parenteral solutions, and monitoring can be found elsewhere (Dworkin and Dworkin, 1990; Dworkin and Dworkin, 2004; Ochala et al., 2011a). Briefly, all experimental group animals were mechanically ventilated, sedated with inhalational isoflurane and pharmacologically post-synaptically paralysed with α-cobratoxin in continuous infusion. Prednisolone 5 mg/kg/day (Prednisolone Alternova, Alternova, skælskør, Denmark), BGP-15 40 mg/kg per day (N-Gene Research Laboratories Inc., New York, United States), and vamorolone 20 mg/kg/day (ReveraGen BioPharma, Rockville, MD, United States) were administered to their respective groups by oral gavage. Experiments were terminated at day 5 or day 10.

### 2.2 Chemically skinned diaphragm muscle fibre preparation

Dissection of diaphragms and fibre bundles for subsequent cryo-storage followed the protocol given in (Corpeno et al., 2014) and (Renaud et al., 2013). Briefly, fibre bundles of ∼50 fibres were manually dissected from the diaphragm muscle in cold relaxing solution (containing 4 mM Mg-ATP, 20 mM imidazole, 7 mM EGTA, 14.5 mM creatine phosphate, 1 mM free Mg^2+^ and ∼1 nM free Ca^2+^, pH adjusted to 7.0 and KCl added to adjust ionic strength to 180 mM), then tied to glass capillaries under slight stretch and chemically skinned in relaxing solution with 50:50 v/v glycerol added for 24 h at 4 °C. An interim treatment with increasing concentrations of sucrose (0.5—2 M) at −20 °C as cryo-protectant followed for 2 weeks, and fibre bundles were then removed from the glass capillary and snap-frozen in liquid nitrogen-chilled propane before long-term storage at −160 °C (Frontera and Larsson, 1997). Cryo-stored samples were transferred between laboratories and on the day prior to experiments, sucrose was sequentially washed out by successive 30 min incubations in decreasing sucrose concentrations before storing in relaxing solution at −20 °C. For ultrastructural *Second Harmonic Generation* (SHG) imaging, bundles were prepared by stretching them out to their full length, pinning them under slight stretch onto a polydimethylsiloxane-coated (PDMS, *Sylgard*, Dow Corning, Wiesbaden, Germany) Petri dish so they were just straightened, followed by fixation with 4% paraformaldehyde in normal PBS and overnight incubation at 4 °C. For dissection of single fibres, the solution was exchanged to normal PBS, and a single fibre was manually tethered from the bundle using two pairs of fine forceps (type 55, Dumont) under a stereomicroscope (Nikon SMZ800N, Nikon, Düsseldorf, Germany). Long single fibre segments were finally transferred to a custom-made optical recording chamber, pushing both ends into opposite streaks of petroleum jelly under slight stretch to position for imaging.

### 2.3 Second Harmonic Generation imaging and quantitative morphometry

For SHG morphometry, an ultra-fast laser-scanning multiphoton microscope (TriMScope II, LaVision BioTec, Bielefeld, Germany) with a mode-locked femtosecond-pulsed Ti:Sa laser (Chameleon Vision II, Coherent, Santa Clara, CA, United States) was used to image the single muscle fibres. The laser was focused into the sample through a ×40 water immersion objective with a numerical aperture of 1.1 (LD C-Apochromat, 40x/1.1, Carl Zeiss, Jena, Germany) and tuned to 810 nm, with a pulse frequency of 80 MHz and average output power of 180 mW. As the SHG signal intensity strongly correlates with the polarization of the incident light (Both et al., 2004), additionally, an adjustable half-wave (λ/2) plate was placed at the back aperture of the excitation objective, to ensure linear light polarization. The polarization angle was individually adjusted for every measured sample through a rotational stage containing the λ/2 plate. On the transmission side, the emitted light was collected by another water immersion objective with a magnification of ×20 and a numerical aperture of 1.0 (W Plan-Apochromat, 20x/1.0, Carl Zeiss, Jena, Germany) and detected by an ultrasensitive transmission photomultiplier tube (H 7422–40 LV 5M, Hamamatsu Photonics, Herrsching, Germany), equipped with a 405/20 nm bandpass filter (CHROMA ET 405/20x, Chroma, Olching, Germany). The imaging parameters were set to 1,024 × 1,024 pixels per image with a corresponding image size of 200 × 200 μm and line scanning frequency of 600 Hz, which results in a lateral physical pixel size of 0.195 μm and a pixel dwell time of 1.41 μs. To extract three-dimensional structural and morphological data from the samples, recording XYZ volumetric image stacks was the imaging procedure of choice. The samples were imaged at three different locations along each fibre to maximize the validity of the extracted features per fibre. The step size in axial direction was set to 1 μm, which yielded, in consideration of the lateral pixel size, a physical voxel size of 0.195 × 0.195 × 1 μm.

### 2.4 Pattern recognition image analysis and quantitative morphometry

Four different parameters were analyzed, (i) SHG signal intensity, (ii) fibre diameter (FD), (iii) Vernier density (VD) and, (iv) Cosine Angle Sum (CAS). The determination of the SHG signal intensity throughout an entire Z-stack of single muscle fibres was performed using a custom-written macro tool called *SHG intensity* within the open-source image analysis software *Fiji*. This tool automatically applies thresholds and exclusively considers signal intensities between 20 and 65,535 a. u. to exclude low-intensity image noise. Afterwards, it consecutively assesses the mean intensity value of each slice and ultimately calculates the average intensity for the entire stack.

As well as for the SHG signal intensity, Fiji was also used to extract the fibres’ FD. A lookup table was applied, and brightness and contrast were adjusted to facilitate manual feature extraction. The largest diameter was measured, using the Straight Line tool, at three different locations across the fibre length. Myofibrillar pattern features, like CAS and VD, were extracted using our previously described algorithm (Friedrich et al., 2010; Garbe et al., 2012). Verniers occur as Y-shaped patterns due to in-register misalignments of otherwise parallel aligned myofibrils and were assessed as Vernier counts per 100 μm^2^ of muscle cross-sectional area, i.e., Vernier density (VD) in #/100 μm^2^ (Friedrich et al., 2010). VDs close to zero indicate an undisrupted, perfectly alternating sarcomere pattern, whereas high VD values are indicative of adjacent myofibrils being out-of-register (Figure 1B). CAS as feature of local myofibril angular orientation distribution was extracted using Equation 1,

$$CAS=\frac{1}{\left|\Omega \right|}\sum_{\left(x,y\right)\in \Omega }\cos\left\{\Phi \left(x,y\right)-median\left[\Phi \left(x,y\right)\right]\right\}$$ (1)

Where $\left|\Omega \right|$ is the number of pixels representing the surface of a fibre, $\Phi \left(x,y\right)$ is the local direction, median [ $\Phi \left(x,y\right)$ ] is the main direction of the fibre, and Ω is the fibre area of all slices of a whole XYZ-stack. While CAS values of zero represent a randomly oriented isotropic ultrastructure, CAS values close to unity indicate a parallel alignment of adjacent myofibrils within the analysed fibre field-of-view (Buttgereit et al., 2014) (Figure 1B).

### 2.5 Statistical analysis

Statistical analysis was performed using the SigmaPlot software version 14 (Systat Software, Erkrath; Germany). Morphometry data of the control and treatment groups were first tested for normal distribution applying Shapiro-Wilk testing. As morphometry data were in general not normally distributed, a two-way analysis of variance (ANOVA) was applied to test for significant differences of data regarding the variables of ICU duration (days 0, 5, 10) and treatment groups (treatment BGP-15, VBP-15, Prednisolone) including interactions between the two variables. To dissect each parameter more thoroughly, one-way ANOVA tests were applied to the data (SHG intensity, FD, CAS, VD) to test for: (i) significant influence of MV + NMBA duration to the untreated MV + NMBA ICU rat group (control, ‘ctrl’), (ii) significant influence of any pharmacological treatment at a given day over the untreated (‘none’) group and (iii) whether any treatment was capable of restoring morphology back to levels of the control group at day 0. (i) is indicative of the prolonged ICU condition (MV + NMBA) affecting cytoarchitecture ‘*per se*’, (ii) is indicative of preventing or ameliorating (i), (iii) is indicative of (ii)’s potency to restore/maintain cytoarchitecture to control levels. Statistical results were considered significant at the *p* = 0.05 level and are expressed as *: *p* < 0.05 for significant, *p* < 0.001 for highly significant and ‘n.s.’ for not significant.

## 3 Results

### 3.1 Quantitative SHG morphometry tracks single fibre atrophy and myofibrillar disorder in diaphragm during ongoing ICU condition and pharmacological intervention

**FIGURE 2:**
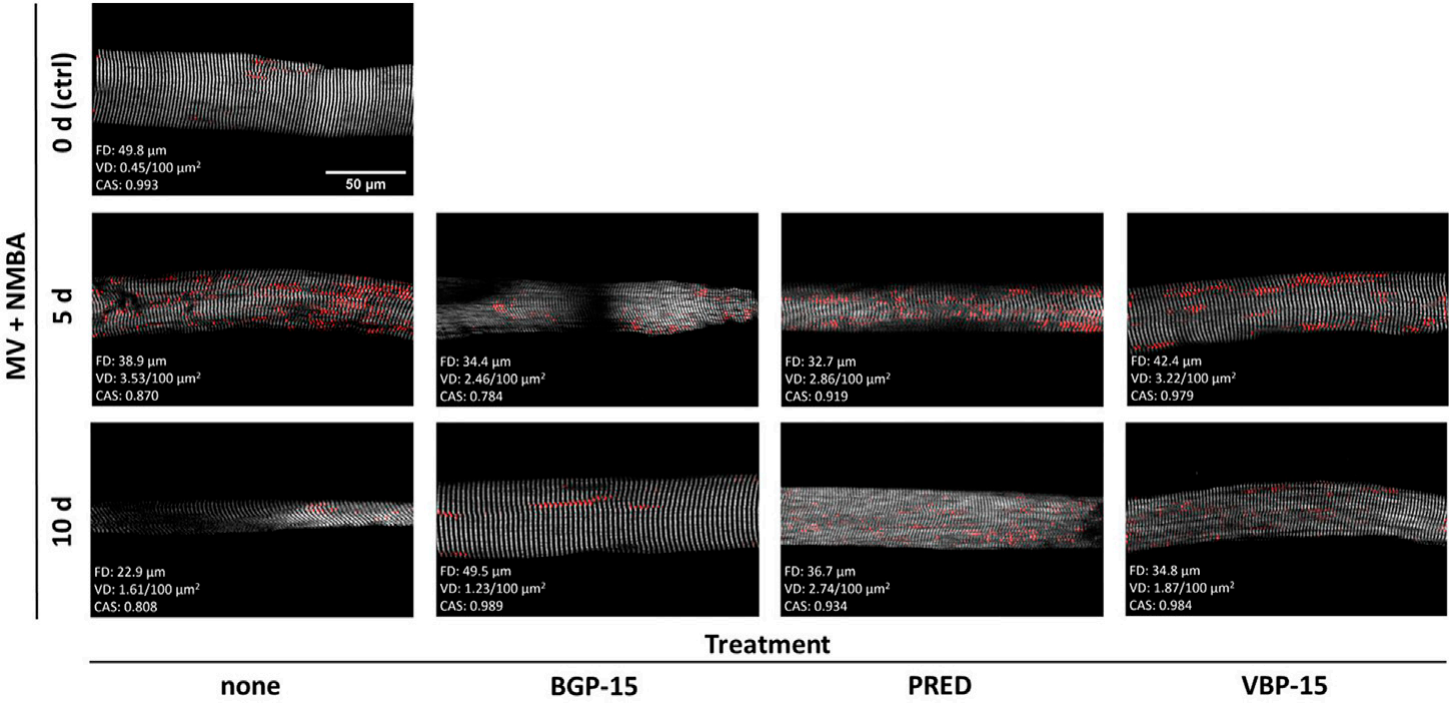
Representative example SHG images with VD as detected by the automated pattern recognition algorithm (red), CAS and fibre diameter (FD) values over the course of the ICU condition (MV + NMBA, treatment: none) and with each of the treatments investigated. Under ICU condition of MV + NMBA, fibre atrophy has already developed after 5 days and is clearly visible in untreated rats after 10 days. In addition, increasing myofibrillar disorder was noted, which appeared to be much less pronounced in the rat diaphragm muscle fibres treated with BGP-15 as compared to VBP-15 and Prednisolone (PRED) treatment. Scale bar applies to all images.

As detailed in the Methods section, we applied *Second Harmonic Generation* (SHG) imaging followed by quantitative morphometry to single muscle fibres from diaphragms of rats subjected to a 10-day ICU-intervention involving controlled mechanical ventilation (MV) and neuromuscular blockade (NMBA) (Figure 1A). Animals belonged to a ‘no pharmacological treatment’ group (control) or either received glucocorticoids (GCs, i.e., Prednisolone), dissociative GCs (VBP-15) or the chaperone co-inducer BGP-15. Figure 2 shows a panel of representative images from each condition at the assessment time points of 5 days and 10 days of interventions compared to the starting point (0 days, ctrl). In the untreated ICU condition (first column), development of single fibre atrophy is already apparent at day 5 and progresses through day 10. This level of atrophy already seems less pronounced in any of the treatment groups, in particular for the BGP-15 group. The quantitative morphometry analysis, providing the cosine angle sum (CAS) and the Vernier density (VD, individually detected verniers labelled in red) suggests a marked myofibrillar disarray as indicated by a low CAS and increasing VD values with ongoing ICU treatment. This disarray seems somewhat prevented by the pharmacological interventions, rather more so by BGP-15 and VBP-15 and to a lesser extent by Prednisolone.

**FIGURE 3:**
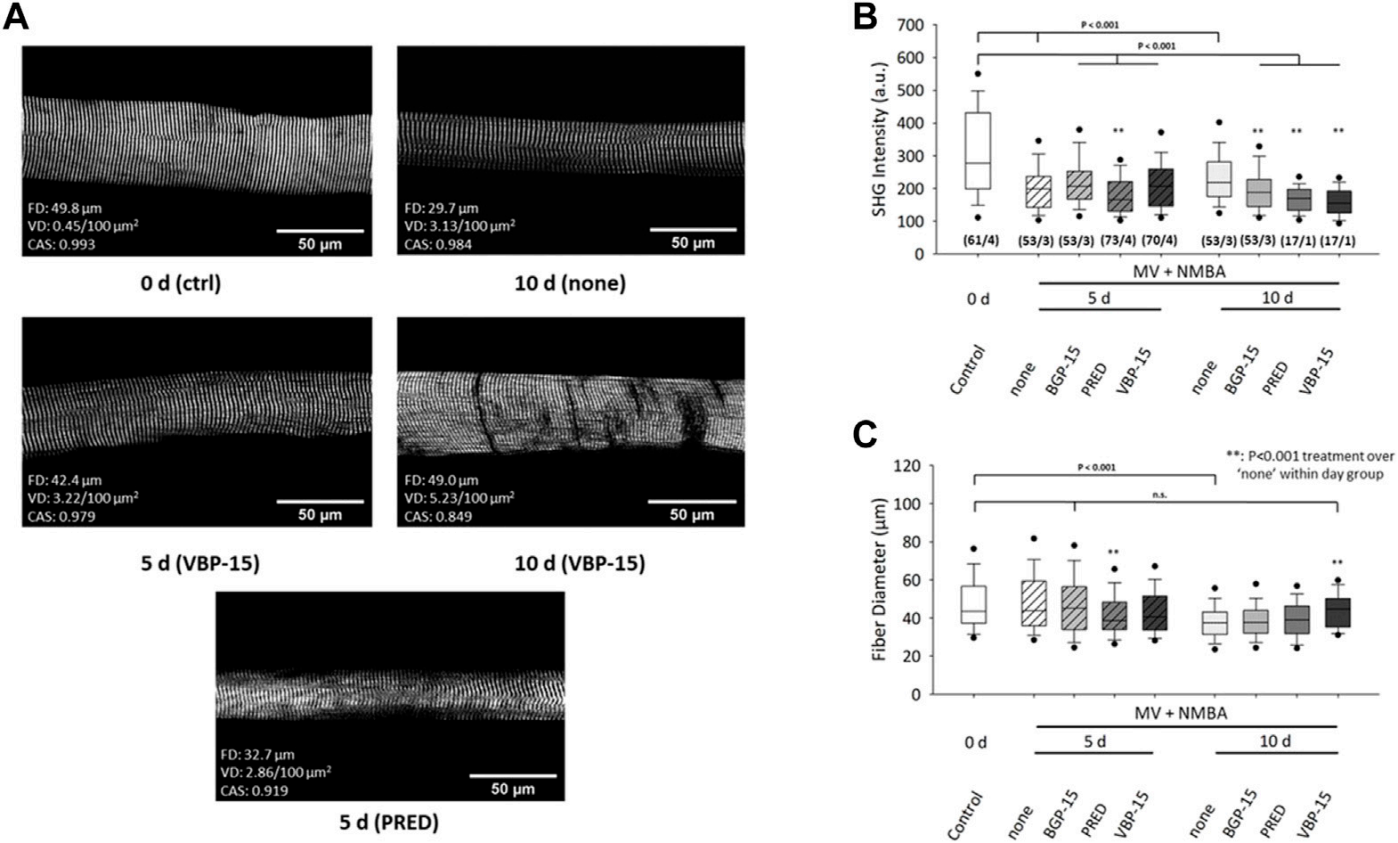
Effects of 10 days MV + NMBA intervention without drug treatment (none) or under treatment with Prednisolone (PRED), VBP-15 or BGP-15 on myosin SHG signal intensities and FDs of single diaphragm muscle fibres. **(A)** example SHG images from a control fibre (ctrl, 0 days) and after 10 days of MV + NMBA showing marked atrophy and faded SHG signal intensity. VBP-15 treatment in a fibre after 5 days and 10 days, implicating potentially reduced atrophy. Prednisolone treatment (5 days) fibre showing a small FD indicative of single fibre atrophy. **(B)** statistical analysis of mean SHG intensities in single fibre images reveals a significant signal decline in untreated ICU rats over the course of 10 days that does not positively respond to any of the treatments. **(C)** FDs confirm a significant decline in untreated animals at day 10 that can be restored to levels similar to controls by VBP-15 (n.s to ctrl) (m/n) (number single fibres/animals).

A statistical analysis of fibre diameters (FD) and mean SHG intensity values obtained from the field-of-view of each individual single fibre for either untreated (‘none’) or pharmacologically treated rats under ICU condition (MV + NMBA) is shown in Figure 3. Ongoing ICU condition results in marked SHG signal loss for all conditions compared to controls at day 0. This means that myosin-II originating SHG signals could not be maintained by any of the pharmacological interventions and in fact even significantly worsened by the drug interventions. This was particularly seen at day 10 while at day 5, worsening was only apparent for Prednisolone whereas BGP-15 and VBP-15 showed a tendency toward slightly improved SHG signal intensities over the untreated ICU-condition, but still being significantly compromised by the ICU-condition itself (Figure 3B). As for the development of fibre atrophy, the ICU condition with no pharmacological intervention resulted in no detectable atrophy at day 5 which then turned into a significant reduction of FD seen at day 10. As for the efficiency of the drug treatments over the untreated ICU-condition (‘none’), Prednisolone promoted fibre atrophy already at day 5 while BGP-15 and VBP-15 showed no effect albeit BGP-15 was capable to preserve FD to levels similar as seen at day 0 (BGP-15 at day 5 not significantly different ‘n.s.’ *versus* control at day 0). This changed for ongoing MV + NMBA intervention with FD being significantly improved at day 10 only by VBP-15 over the untreated ICU group (‘none’) and even restoring those FD values to levels similar to the day 0 control group (VBP-15 day 10 not significant *versus* control at day 0).

**FIGURE 4:**
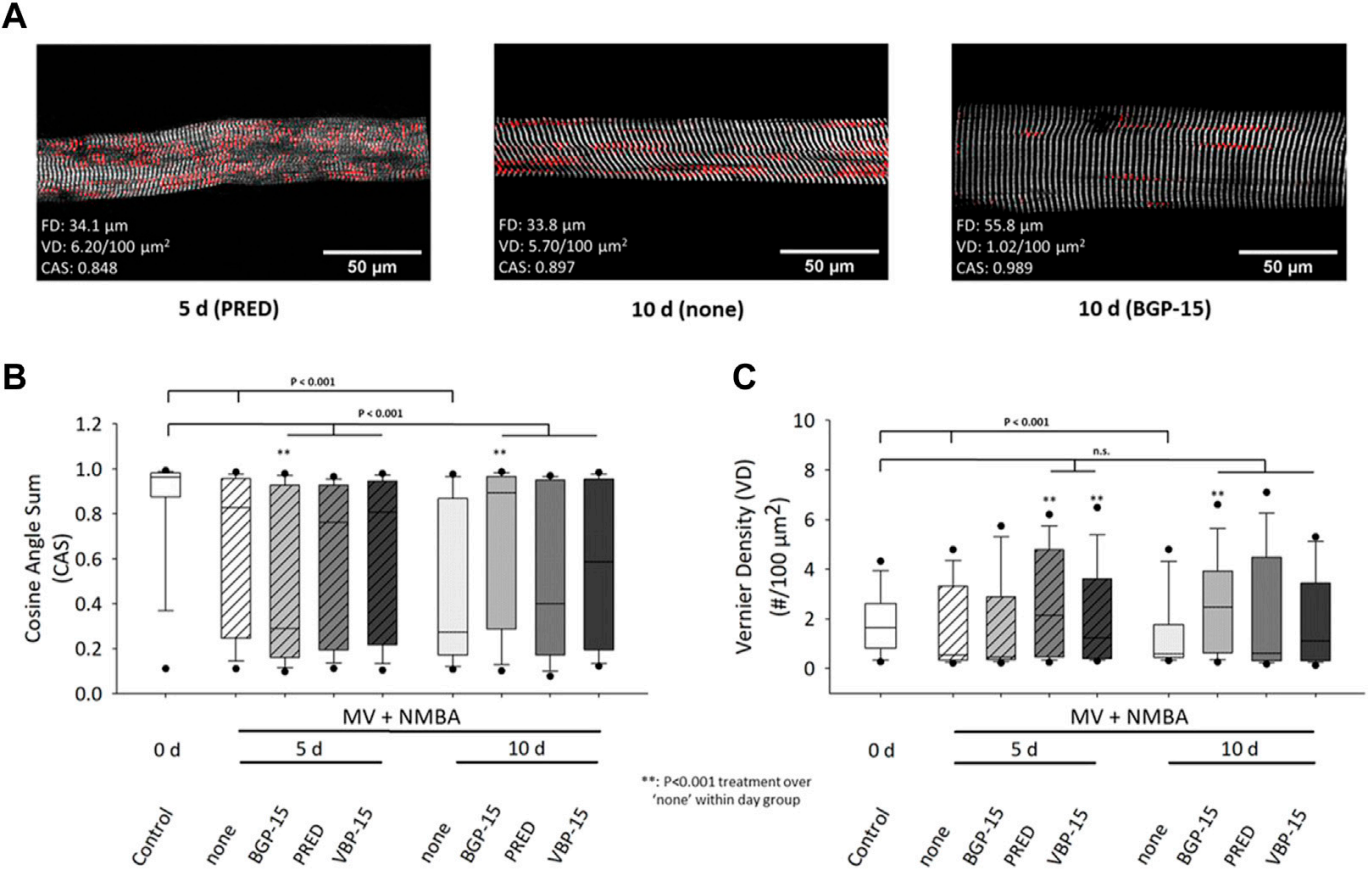
Effects of a 10 days MV + NMBA intervention without drug treatment (none) or applying Prednisolone (PRED), VBP-15 or BGP-15 treatment on single diaphragm muscle fibres’ CAS and VD ultrastructural parameters. **(A)** example SHG images from a single fibre after 5 days Prednisolone treatment (left), after 10 days of no treatment (middle) and following BGP-15 treatment (right) alongside with their analysed diameter, CAS and VD values. The images already suggest a marked disarray of myofibrillar lattice during the course of ICU condition (MV + NMBA) that is largely prevented in the BGP-15 but not the Prednisolone-treated fibre. **(B)** statistical analysis of mean CAS values in single fibre images reveals a significant decline in untreated ICU rats over the course of 10 days that does partially respond to the worsening of CAS at 10 days by the pharmacological interventions (in particular for BGP-15) but is not able to restore control levels (all treatments still being significantly different to control). **(C)** VD values significantly decline during the course of untreated ICU condition (treatment: none) but are mostly kept within the control level range for all treatments, in particular after 10 days.

The statistical analyses after 10 days under ICU condition (MV + NMBA) and pharmacological interventions affecting myofibrillar cytoarchitecture (CAS, VD) of single diaphragm fibres are shown in Figure 4. ICU condition resulted in a significant decrease in CAS values already at day 5 and further dropped until day 10 (Figure 4B). Within each follow-up bin (day 5, day 10), the glucocorticoids Prednisolone and VBP-15 did not have any discernible effect on CAS compared to the ICU treatment itself. Only BGP-15 showed some differential effect, with a significantly lowered CAS at day 5 but then significantly increased CAS at day 10. More importantly, none of the pharmacological interventions was able to restore CAS values back to or preserving them at the control values of day 0.

For the analysis of VD values for the given conditions (Figure 4C), ICU treatment itself (MV + NMBA) resulted in a consistently significant decline in VD values in the untreated (‘none’) group through day 10. While the comparison within treatment days showed some inconsistent behaviour compared to the untreated ICU condition (MV + NMBA), it is noteworthy that all treatments were capable to restore or maintain VD values to values similar as in the control group at day 0, except for BGP-15 at day 5 which was significantly reduced there (but returned to control levels at day 10).

## 4 Discussion

The aim of our current study was to (i) unravel the time course of subcellular cytoarchitectural remodelling of the myofibrillar lattice array in the diaphragm during ongoing mechanical ventilation and neuromuscular blockade in a rat model of VIDD and (ii) to clarify whether treatment regimes, involving corticosteroids (classical Prednisolone or dissociative steroid VBP-15) or BGP-15 chaperone co-inducer, would be able to maintain subcellular cytoarchitecture, therefore, providing a pharmacological ‘splint’ to prevent weakness imprinted by structural myofibrillar disorder in VIDD.

### 4.1 Time course of VIDD-related myofibrillar remodelling and atrophy during prolonged MV and NMBA

Although mechanical ventilation is a life-saving ICU intervention in conditions of impaired intrinsic pulmonary function, the development of diaphragm atrophy and contractile dysfunction leading to weaning failure and prolonged ICU treatment has been proposed to start as early as 24 h after initiation of MV in patients (Powers et al., 2013; Spiesshoefer et al., 2023) and some animal models (Zhang et al., 2019). Muscle fibre atrophy is one of the hallmarks in critical illness myopathy (CIM) (Friedrich et al., 2015; Friedrich et al., 2017; Akkad et al., 2019) and VIDD in the diaphragm (Corpeno Kalamgi et al., 2016; Matecki et al., 2016; Salah et al., 2016; Hyatt and Powers, 2020). However, both time courses and underlying mechanisms seem to be distinct. In limb muscles, myosin post-translational modifications precede the preferential myosin loss while actin content is spared or less affected, as seen by a reduced myosin:action ratio in single muscle fibres already as early as 5 days of ICU treatment in the same experimental ICU-rat model used here (Ochala et al., 2011a; Friedrich et al., 2017; Akkad et al., 2019; Cacciani et al., 2020). In contrast, single fibre cross-sectional area was maintained during the first initial 4 days of controlled mechanical ventilation and neuromuscular blockade, followed by a progressive decline in fibre size (CSAs ∼30% smaller after 5–8 days and ∼50% smaller after 9–14 days) as compared to control diaphragm fibres (Corpeno et al., 2014; Salah et al., 2016). This is also confirmed by our label-free SHG assessment of FDs, showing unchanged diameters at 5 days of MV + NMBA but significantly reduced ones at 10 days of MV + NMBA compared with controls at day 0. The initially preserved fibre CSA was also seen in a porcine ICU model where diaphragm from piglets undergoing MV + NMBA did not show any alterations in CSA, myosin heavy chain isoforms or contractile protein content at 5 days of intervention (Ochala et al., 2011b). However, due to the demanding experimental nature of the ICU-pig model, no longer intervention times could be assessed in that study. Nevertheless, atrophy in the diaphragm in VIDD seems to be governed by slower kinetics than in limb muscles in CIM also across species, and this may also reflect their differential underlying mechanisms.

In limb muscles, increased proteolytic degradation pathways and downregulation of genes related to contractile, regulatory and mitochondrial protein synthesis are supposed to mainly account for the predominant loss of myosin (Norman et al., 2006; Llano-Diez et al., 2011; Akkad et al., 2014; Friedrich et al., 2017). In the diaphragm, on the other hand, oxidative stress with ROS production (Powers et al., 2002; Zergeroglu et al., 2003), post-translational modifications (e.g., carbonylation, SUMOylation) and intracellular lipid accumulation, all appear to play a much more important role in the response to MV + NMBA with equally declining myofibrillar protein contents, transcriptional downregulation of actin and myosin synthesis and thus, preservation of myosin:actin ratios over at least 14 days in the rat-ICU model (Corpeno et al., 2014). These effects seem to result in a more slowly developing global atrophy from day 4–5. It is important to note that the rat model involved in the aforementioned and the current study was not subjected to any additionally confounding conditions of critical illness (e.g., sepsis, trauma, *etc.*) but rather reflects the setting of VIDD as a consequence of the ICU-treatment (MV + NMBA).

Muscular weakness and fatigue are the most prominent symptoms resulting from ICU-interventions, both for limb muscles in CIM and the diaphragm in VIDD (Larsson et al., 2000; Friedrich et al., 2015; Larsson and Friedrich, 2016). The question of whether fibre atrophy was the sole reason for the observed muscle weakness, has often been addressed in the literature. In general, many studies have found the ‘quality of contraction’ to be compromised beyond the degree of single fibre atrophy as judged from marked decline in specific (cross-sectional area normalised) force in CIM and VIDD (Larsson et al., 2000; Ochala et al., 2011b; Aare et al., 2013; Akkad et al., 2019; Shrager et al., 2021). Although critical illness results in a plethora of dysregulations of genes responsible for protein turnover in muscle, for instance for cytoskeletal and sarcomeric genes (Aare et al., 2013), also changes in the signaling network have been held responsible for diaphragm weakness (Tang and Shrager, 2018). Of particular interest has been oxidative stress-related endoplasmic reticulum dysfunction (Li et al., 2022), resulting in leaky ryanodine receptors (Matecki et al., 2016) with consecutively disturbed Ca^2+^ homeostasis in humans and animal models of VIDD (Hyatt and Powers, 2020). In MV + NMBA treated rats, the decline in diaphragm function preceded atrophy, and at the end of 2 weeks of ICU treatment, residual diaphragm muscle function was <15% of control levels (Corpeno et al., 2014). Specific force was already 25% lower between 6 h and 4 days of intervention, and progressively declined for longer durations (50% lower after 5–8 days, 70% lower after 9–14 days; Corpeno et al. (2014)). X-ray diffraction experiments carried out in that same study showed that structural integrity of diaphragm single muscle fibre myofibrils was maintained over the whole time course of MV + NMBA in rats, and intensity ratios of X-ray reflections in the relaxed state did not change significantly, indicating that the actin-myosin binding interaction was still possible in an ATP-dependent manner even after 14 days of ICU intervention (Corpeno et al., 2014). What was even more intriguing was that a significant increase in the filament lattice spacing was detected with ongoing ICU intervention, suggesting degradation of some of the linkage proteins holding the myofilaments together, e.g., titin (Corpeno et al., 2014). This already points towards a structural component to the ICU-induced weakness in VIDD, as the 70% lower specific force in the 9–14 days group could not be solely explained on the basis of the ∼20% lower myosin quantity.

What could not be derived from those aforementioned studies so far, was how the 3D sterical aspect of myofibrillar lattice geometry was affected by the ICU intervention. This is where our current SHG-based morphometry analyses add valuable and novel insights into structure-function relationships in VIDD. Strikingly, although there was a marked variability in SHG morphometry parameters, in particular for cosine angle sums (CAS), statistical analysis revealed significantly reduced CAS values for single diaphragm fibres from MV + NMBA treated rats compared to control levels (median ∼0.97), progressing from 5 days (median ∼0.85) to 10 days (median ∼0.25). As shown in our previous work, CAS values close to 1 reflect a highly parallel alignment of myofibrils, while lower CAS values are indicative of less ordered fibrillary networks, including local tilts, twists and angular deviations from the main fibre axis (Friedrich et al., 2010; Buttgereit et al., 2013; Buttgereit et al., 2014).

In a chronic muscle degeneration-regeneration model of the *mdx* mouse, we previously could directly establish the relationship between CAS and predicted force output on the single fibre level in *extensor digitorum longus* (edl) muscle using our in-house engineered *MechaMorph* system that allows simultaneous SHG imaging and force recordings in single fibres (Schneidereit et al., 2018). From those calibrations, it becomes apparent that a CAS of ∼0.85 is expected to produce already only 50% of the levels seen in healthy wt fibres (see Figure 4 in Schneidereit et al. (2018)), and that the much lower values at 10 days of MV + NMBA are well compatible with even much less force as seen in (Corpeno et al., 2014). However, for a precise correlation, simultaneous SHG and force recordings would have to be performed using diaphragm muscle fibres which has to be addressed in future studies. Interestingly, unlike in the *mdx* limb muscle single fibres, the VD values showed an opposite behaviour in the diaphragm from ICU rats, with median values being significantly lower as compared to the controls, while in *mdx* edl fibres, VDs were always larger in *mdx* compared with wt fibres (Friedrich et al., 2010; Schneidereit et al., 2018). This may point towards a different remodeling mechanism which seems obvious from the predominantly necrosis-driven remodeling patterns in muscular dystrophy as opposed to the more prominent changes in posttranslational protein modifications (PTMs), imbalance in protein synthesis and proteolysis and impaired chaperone regulation in VIDD (Salah et al., 2016). A comparison with the CAS-VD-force calibrations given in Schneidereit et al. (2018) (their Figure 4), also explains why the force output probably is still to some extent held up despite CAS levels around 0.25 suggesting a diminished force production: lower VD values around 0.5 (median in our MV + NMBA 5 days and 10 days groups) would still be associated with a roughly 20% higher force production as compared to fibres showing VD values around 1.5 (median in the control group at 0 days).

Of note, the overall significant decline in SHG intensity seen in all VIDD groups vs control regardless of treatment is suggestive of a decline in myosin content that does, however, not allow conclusions toward preferential myosin proteolysis as the SHG signal does not trace back to actin filaments as signal source, only myosin. Previous studies using single fibre SDS-PAGE showed that myosin:actin ratios in diaphragm were mostly preserved during 14 days of MV (Corpeno et al., 2014). Therefore, a global decrease in actin and myosin content would also be expected to be seen as a decline in SHG signal, as shown here. The add-on value of the SHG morphometry thus, lies more in the myofibrillar orientation analysis (CAS, VD) as performed here. The SHG signal intensity on its own is probably of less diagnostic value and may be combined with single fibre myosin typing following SHG imaging in the future.

### 4.2 Small molecule treatment with VBP-15 or BGP-15 compared to Prednisolone during the course of MV + NMBA-induced VIDD

Glucocorticoids (GCs) have been widely used to control systemic inflammation such as in systemic inflammatory response syndrome (SIRS) and other intensive care conditions (Cavaliere et al., 2004). They play an important role in survival and recovery in ICU patients by counteracting inflammation and ischaemia, thus reducing the occurrence of organ failure and duration of mechanical ventilation (Annane et al., 2006). However, classical GCs, like Prednisolone, are also known to be responsible for the adverse outcome on skeletal muscle by promoting myofibrillar protein degradation (Schakman et al., 2013; Akkad et al., 2019). Therefore, searching for alternative compounds, i.e., small molecules, to control the pro-inflammatory aspect of ICU-treatment while avoiding their myotoxic component, has become a major focus.

Vamorolone (VAM or VBP-15) belongs to a class of dissociative GCs reported to selectively activate anti-inflammatory pathways while minimizing activation of ‘pro-myopathy’ pathways thus, dissociating the two branches of actions seen in classical GCs (Reeves et al., 2013). As steroid receptor-modulating compound, VBP-15 has already shown beneficial effects in muscular dystrophy as well as inflammatory myopathies (Heier et al., 2019) (Akkad et al., 2019). In a MV + NMBA study in rats comparing the effects of the glucocorticoid Prednisolone or VBP-15 on limb muscle function at day 5 of ICU intervention, both drug treatment regimens produced reduced atrophy and single fibre weakness in the *soleus,* but only VBP-15 was effective in compensating atrophy and specific force loss in edl single fibres, while Prednisolone was detrimental to both parameters and even worsened specific force loss at day 5 seen under MV + NMBA only (Akkad et al., 2019). This was paralleled by VBP-15 being efficient in lowering MuRF1 and atrogin-1 transcription levels in the *soleus* compared to the MV + NMBA groups without (8-fold and 3- to 4-fold increase, respectively) and Prednisolone (20-fold and 3- to 4-fold increase, respectively). In contrast, E3 ligases were not efficiently lowered by VBP-15 in the fast-twitch edl (Akkad et al., 2019). In diaphragm muscle, to our knowledge, no study has yet assessed the functional consequences of VBP-15 in ameliorating VIDD. However, as adult human diaphragm is composed of a balanced composition of slow and fast myosin isoforms (Mercadier et al., 1998), VBP-15 is expected to also functionally ameliorate diaphragm performance in the MV + NMBA setting which awaits experimental confirmation.

From a structural point of view, our morphometry analyses already point towards a potent effect of VBP-15 to also compensate for atrophy in the diaphragm during ongoing MV + NMBA, with significantly larger FDs at day 10 over untreated rats and rats treated with Prednisolone. This compensation was even capable to restore levels of FDs to the control level. As for myofibrillar remodeling, VBP-15 did not have any discernible impact on CAS levels which still remained significantly below control values and similar to the reduced levels in the MV + NMBA groups at day 5 and day 10. In contrast, VBP-15 seemed to potently restore VD levels back to day 0 control values, an effect that was mostly shared among all the drug treatment groups (Prednisolone, VBP-15 and BGP-15).

BGP-15 belongs to another class of small molecules and acts as a heat shock protein (HSP) chaperone co-inducer that has been shown to improve muscle architecture, strength and contractile function in severely compromised diaphragm in dystrophic *mdx* mice (Gehrig et al., 2012), protected against heart failure and atrial fibrillation (Sapra et al., 2014) and also alleviated ventilation-induced diaphragm dysfunction in the MV + NMBA rat model (Salah et al., 2016). BGP-15 also blocks TNF-α-induced pro-inflammatory pathways, improves mitochondrial efficiency and reduces reactive oxygen species (ROS) production (Henstridge et al., 2014), all of which would otherwise contribute to the posttranslational myofibrillar protein modifications seen in VIDD (Salah et al., 2016). This was documented in their study of 10 days controlled mechanical ventilation in rats where BGP-15 treatment improved diaphragm single muscle fibre force-generating capacity by more than 100% and reduced myosin PTMs (Salah et al., 2016). However, from a structural point, systemic administration of BGP-15 did not significantly influence the decline in single fibre CSA of ∼50% that was seen after 10 days of MV + NMBA (Salah et al., 2016). Interestingly, in another age-related study of BGP-15 effects in VIDD in rats, BGP-15 only restored force capacity in single diaphragm fibres alongside increased HSP72 expression in young (7–8 months) rats while having no positive effect in old (28–32 months) rats (Ogilvie et al., 2016). This may be explained by a strongly decreased HSP72 expression in some muscle types (i.e., fast-twitch muscle) in aged animals (Locke, 2000) that may not be completely brought back to levels seen in young animals by BGP-15 treatment.

Since upregulation of HSP72 maintains fibre integrity and facilitates muscle regeneration (Senf, 2013), impaired HSP72 expression is in line with deleterious effects on muscle structure and function seen for mitochondrial morphology in diaphragm EM images of MV + NMBA treated rats (Salah et al., 2016). Similarly, disturbed sarcomeric uniformity affecting regulation of muscle contraction is also a consequence of PTM-induced perturbations in the myosin rod domain (Cammarato et al., 2011) in MV + NMBA and is expected to be reduced by BGP-15 treatment (Salah et al., 2016). As such, again, our study provides a first direct indication of how chaperone-induction in MV + NMBA intervention can restore some of the myofibrillar architectural disarray seen in VIDD. Confirming the results from Salah et al. (2016), our SHG morphometry revealed no positive effects of BGP-15 on the fibre atrophy seen in the mechanically ventilated rats. However, BGP-15 was capable of significantly restoring/maintaining CAS levels compared to the ICU group (‘none’) at 10 days of MV + NMBA intervention. Although this effect was still only partial and behind the control levels (day 0), it already points towards BGP-15 being able to maintain or restore the angular disarray of myofibrils in single diaphragm muscle fibres of rats subjected to ongoing MV + NMBA regimens, which is consistent with a high force output (Schneidereit et al., 2018). The effects of BGP-15 on axial lattice order (Vernier densities, VD) were comparable to those of VBP-15 but showed a lag of 5 days.

Nevertheless, it seems that both compounds are capable to restore myofibrillar order and thus, force generation capacity in mechanically ventilated diaphragm in the ICU setting, with a higher emphasis of BGP-15 restoring parallelism of myofibrils while VBP-15 shows more emphasis on axial lattice order.

## 5 Conclusion

In summary, our study confirms several aspects of two previously beneficially proven small molecules, VBP-15 and BGP-15, on single fibre diameters and atrophy in diaphragm from rats undergoing mechanical ventilation and pharmacological neuromuscular silencing up to 10 days of ICU treatment. Although both compounds were tested separately showing differential positive effects, a combined therapy approach would be the next logical step for future studies. Nevertheless, our study is, to the best of our knowledge, the very first one to provide a quantitative single fibre morphometry approach to VIDD and therapeutic interventions employing label-fee Second Harmonic Generation imaging. An important goal for future studies would therefore, also be to establish the direct structure-function relationships in single VIDD fibres using our previously reported *MechaMorph* metrology (Schneidereit et al., 2018).

## Data Availability

The raw data supporting the conclusion of this article will be made available by the authors, without undue reservation.
